# The relationship between ultrasound findings and thyroid function in children and adolescent autoimmune diffuse thyroid diseases

**DOI:** 10.1038/s41598-021-99016-2

**Published:** 2021-10-05

**Authors:** Ji Eun Park, Sook Min Hwang, Ji-Young Hwang, Jin Hee Moon, Ik Yang, Ji Young Woo, Hye Jin Lee

**Affiliations:** 1 Department of Radiology, Ajou University Hospital, School of Medicine, Ajou University, Suwon, Korea; 2 Department of Radiology, Kangnam Sacred Heart Hospital, Hallym University College of Medicine, Singil-ro, Yeongdeungpo-gu, Seoul, 07441 Korea; 3 Department of Pediatrics, Kangnam Sacred Heart Hospital, Hallym University College of Medicine, Seoul, Korea

**Keywords:** Diseases, Endocrinology

## Abstract

To evaluate the association between thyroid echogenicity and heterogeneity seen on ultrasonography (US) and thyroid function in pediatric and adolescent populations with autoimmune diffuse thyroid diseases (AITD). From 2000 to 2020, we reviewed thyroid ultrasound (US) images and thyroid function statuses in 133 children and adolescent AITD patients. Our review of the images focused on decreased echogenicity and heterogeneity, which were classified into four grades. Among patients with overt hypothyroidism or overt hyperthyroidism, 94.2% (65/69) showed a US grade of 3 or 4. In patients with subclinical hyper/hypothyroidism or euthyroidism, 45.3% (29/64) showed grades 1 or 2. There were no overt hyper/hypothyroidism patients with US grade 1. When we compared US grades according to thyroid status, more severe thyroid dysfunction was significantly associated with higher US grade (*p* = 0.047). Thyroid stimulating hormone (TSH) level differed significantly according to US grades when we evaluated hyperthyroid (*p* = 0.035) and hypothyroid (*p* = 0.027) states independently. 11 patients showed both US grade and thyroid function status changes on follow-up US. In children and adolescent AITD patients, there was an association between decreased echogenicity and heterogeneity on US and thyroid dysfunction.

## Introduction

A wide spectrum of conditions such as deficiency of the iodine that is required to produce thyroid hormones, diffuse thyroid diseases, and thyroid nodules can lead to morphologic changes in the thyroid gland^1,2^. The most common category of diffuse thyroid disease comprises autoimmune thyroid diseases (AITD), including Hashimoto’s thyroiditis (HT) and Graves’ disease (GD)^2^. These chronic disorders are characterized by an intolerance to self-thyroid antigens and have a prevalence of 9.6% and 1.3% in children and adolescents, respectively^3^.

In managing AITD, biochemical parameters including thyroid function and antibody levels are checked, and US is usually performed to check for parenchymal abnormalities or emergence of focal lesions^1^. Several previous studies reported US findings of thyroiditis and an association between thyroid gland echogenicity and thyroid function^2,4–10^. Decreased echogenicity of the thyroid gland on US is associated with overt hypothyroidism^4,10^, and changes in echogenicity have been reported in subclinical hypothyroidism as well^5–9^. Another general population study found that thyroid gland heterogeneity on US is related to thyroid dysfunction regardless of whether it is accompanied by hypoechogenicity^5^.

To the best of our knowledge, no studies have evaluated the relationship between US thyroid features, such as decreased echogenicity and heterogeneity, and thyroid function in child and adolescent patients with AITD, including HD and GD. The aim of this study was to evaluate the association between thyroid echogenicity and heterogeneity seen on US and thyroid function in children and adolescents with AITD.

## Materials and methods

### Patients and thyroid function analysis

This retrospective study was approved by the Institutional Review Board of Kangnam Sacred Heart Hospital, all methods were performed in accordance with the relevant guidelines and regulations and the requirement for informed patient consent was waived. At our institution, 372 child and adolescent patients underwent thyroid US between January 2000 and March 2020.

Among the 372 patients, 239 were excluded for the following reasons: 95 did not meet the AITD diagnostic criteria; 85 showed no parenchymal abnormality on US scan; 29 patients were diagnosed with congenital hypothyroidism; 18 showed focal lesions on US with benign cytology results; nine had focal lesions with malignant pathology results; one patient had thyroid gland aplasia; one patient had an ectopic thyroid gland; and one patient developed thyroid gland hypoplasia after abscess formation due to a fourth branchial cleft cyst (BCC). Finally, 133 patients were included in this study. AITD diagnosis was made according to clinical history, symptoms, thyroid function test (TFT) results, and confirmation of antithyroid peroxidase autoantibodies (TPOAb) or serum TSH receptor antibodies (TRAb).

Laboratory data obtained from patient medical records were retrospectively evaluated. Serum concentrations of free thyroxine (fT4) and thyroid stimulating hormone (TSH) were measured using immunoradiometric kits (RIAKEY; Shin Jin Medics, Seoul, Republic of Korea). The serum levels of triiodothyronine (T3), antithyroglobulin antibodies (TGAb), TPOAb, and TRAb were measured using radioimmunoassay kits (Brahms DYNOTest; Diagnostica GmbH, Berlin, Germany). The normal serum ranges for these biomarkers are: fT4: 0.70–1.80 ng/dL (9.01–23.2 pmol/L), TSH: 0.4–4.1 mIU/L, and T3: 87–184 ng/dL (1.34–2.83 nmol/L).

Thyroid function was subdivided into five statuses: overt hypothyroidism (1), subclinical hypothyroidism (2), euthyroidism (3), subclinical hyperthyroidism (4), and hyperthyroidism (5). Overt hypothyroidism (1) was defined by low concentrations of fT4 and elevated serum concentrations of TSH. Subclinical hypothyroidism (2) was defined as elevated serum TSH and normal fT4. Normal TSH, fT4, and T3 levels were defined as euthyroidism (3). Subclinical hyperthyroidism (4) was defined as low serum TSH level and normal fT4 level. Overt hyperthyroidism (5) was defined as elevated concentrations of fT4 and low serum concentrations of TSH. Thyroid hormone levels were checked within one month before the US examination. Antibody levels were evaluated within a month before and after US examination.

We additionally analyzed relationship between US grades and thyroid function status for HT separately from GD. HT was defined when there was elevation of TGAb or TPOAb and GD was defined when there was elevation of TRAb.

### US evaluation

**Figure 1:**
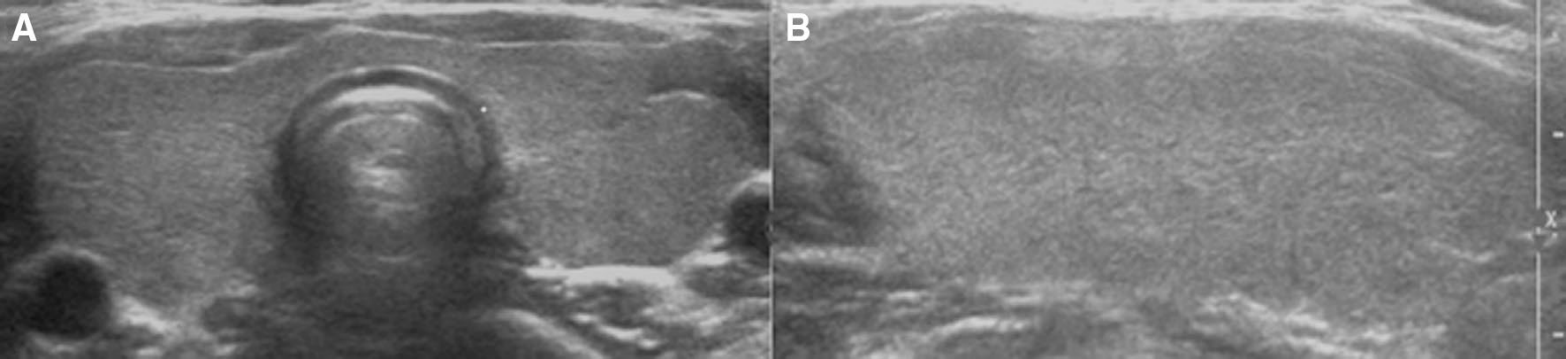
Transverse (**A**) and longitudinal (**B**) US images of G1: diffusely enlarged thyroid gland with normal echogenicity, similar to a normal thyroid gland and hyperechoic compared with the anterior strap muscle (asterisk) without heterogeneity.

**Figure 2:**
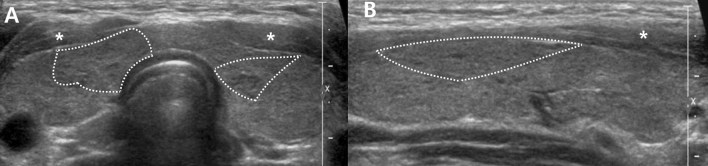
Transverse (**A**) and longitudinal (**B**) US images of G2: diffusely enlarged thyroid gland with heterogeneity involving less than one third of the thyroid gland (area with dashed lines), while the rest of the gland shows normal echogenicity.

**Figure 3:**
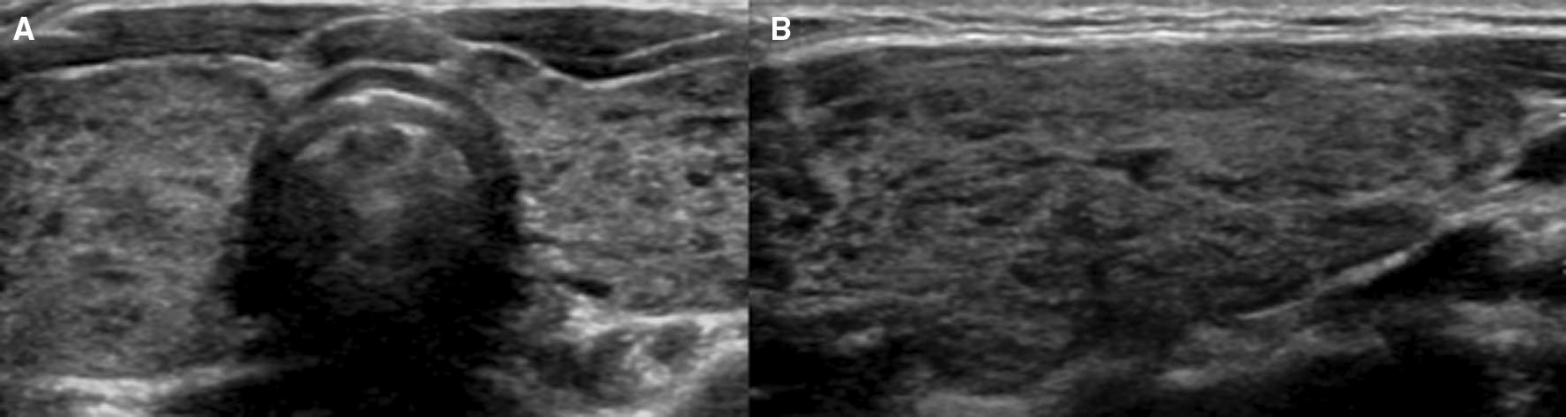
Transverse (**A**) and longitudinal (**B**) US images of G3: diffusely enlarged thyroid gland with heterogeneity involving more than one third of the gland while the rest of the gland shows isoechogenecity compared with the anterior strap muscle.

**Figure 4:**
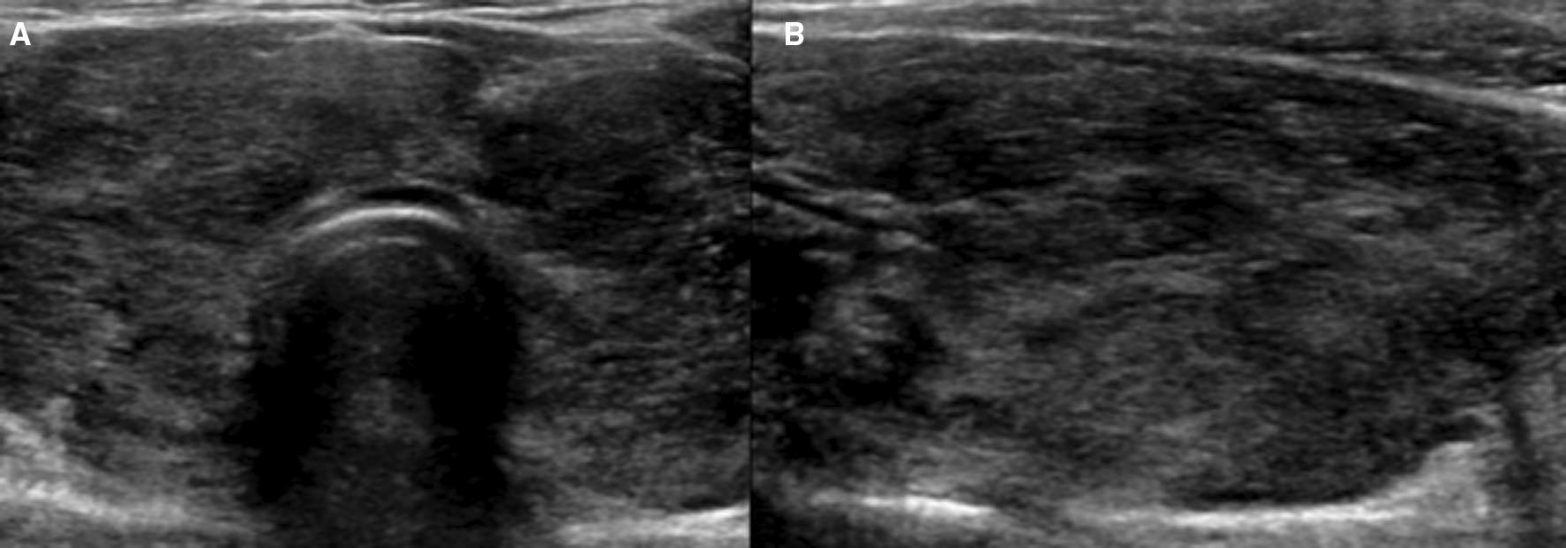
Transverse (**A**) and longitudinal (**B**) US images of G4: diffusely enlarged thyroid gland with diffuse heterogeneity involving more than one third of the gland (area with dashed lines) with marked hypoechogenicity that is more hypoechoic than the anterior strap muscle (asterisk).

US evaluations were performed using a 5–12 MHz linear array transducer (IU22 US or HDI 5000; Philips Healthcare or LogiQ E9; GE Healthcare). One of four radiologists whose experiences with thyroid imaging ranged from 10 to 18 years performed US examinations. Two radiologists with 10 and 11 years of experience in thyroid imaging reviewed the US findings and reached a consensus for each case. The reviewers were blinded to patient hormonal status. The US patterns were classified into four grades according to hypoechogenicity and heterogeneity degrees. Hypoechogenicity was estimated by comparing with the echogenicity of the anterior strap muscle, and heterogeneity was defined as any region with an unclear boundary showing a different echogenicity from other parts of the gland. Grades were as follows: Grade 1 (G1) = diffusely enlarged thyroid gland with normal echogenicity (similar to a normal thyroid gland and hyperechoic to the anterior strap muscle) without heterogeneity (Fig. 1); G2 = diffusely enlarged thyroid gland with heterogeneity involving less than one third of thyroid gland, while the rest of the gland shows normal echogenicity (Fig. 2); G3 = diffusely enlarged thyroid gland with heterogeneity involving more than one third of the gland, while the rest of gland shows isoechogenicity compared with the anterior strap muscle (Fig. 3); and G4 = diffusely enlarged thyroid gland with diffuse heterogeneity involving more than one third of the gland with marked hypoechogenicity that is more hypoechoic than the anterior strap muscle (Fig. 4).

### Statistical analyses

Statistical analyses were performed using SPSS software (SPSS, version 23.0; SPSS, Chicago, IL, USA). Associations were considered significant if the *P* value was < 0.05. Descriptive data are summarized as means ± standard deviations (SD) or numbers of individuals (percentage) with a condition. Statistical differences were compared using Fisher’s exact test for categorical variables. For continuous variables, the Kruskal–Wallis test was used to compare across US grade levels for thyroid function status and antibody titer after testing for normality and equivalent variance.

## Results

### Clinical profiles and laboratory data

**Figure 5:**
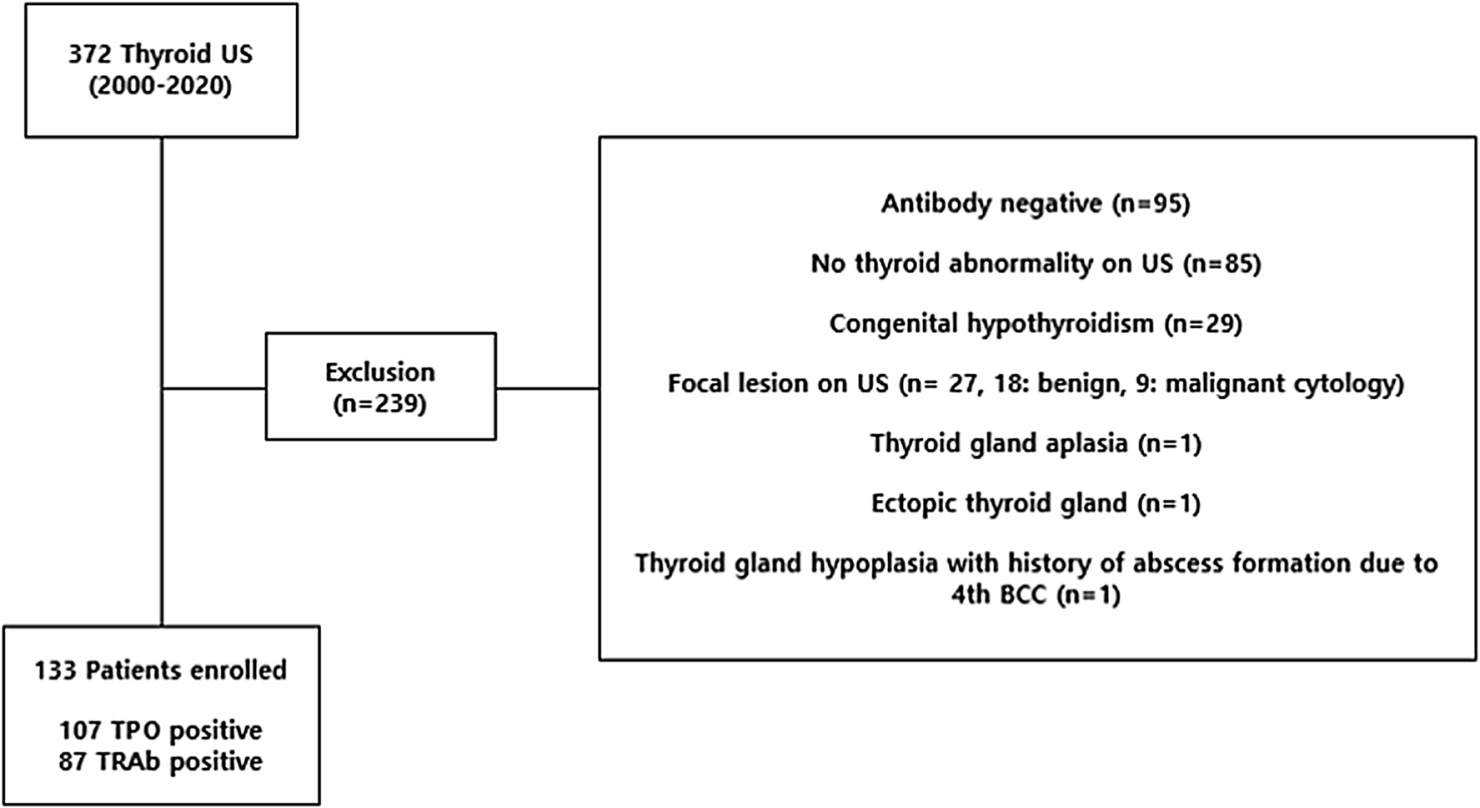
Flowchart of overall study population enrollment.

The mean patient age was 15 ± 3.8 years (range 5–19 years), with 102 (76.7%) females and 31 (23.3%) males. TPOAb levels were evaluated in 107 patients and TRAb levels were evaluated in 87 patients (Fig. 5). There were three patients with simultaneously increased TPO and TRAb.

### US grades and thyroid gland functional status

**Figure 6:**
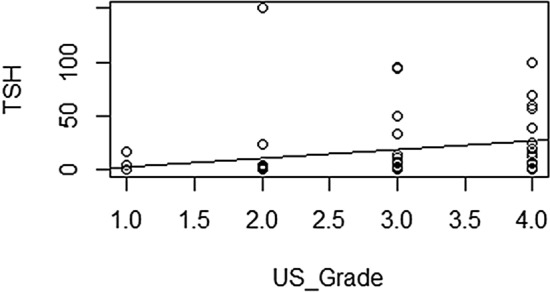
Graphic presentation of linear positive correlation of the TSH level and the severity of US grades in hypothyroidism. (R software, ver. 4.0.4; R Foundation for Statistical Computing, Vienna, Austria).

**Figure 7:**
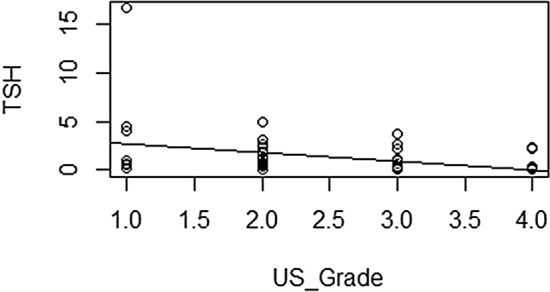
Graphic presentation of linear negative correlation of the TSH level and the severity of US grades in hyperthyroidism. (R software, ver. 4.0.4; R Foundation for Statistical Computing, Vienna, Austria).

Table 1 summarizes the relationship between US grades and functional thyroid status. Eight (6%), 25 (18.9%), 45 (33.8%), and 55 (41.3%) patients were classified into G1, G2, G3, and G4, respectively. Among G1 and G2 patients, 0 (0/8, 0%) and 1 (1/25, 4%) had overt hypothyroidism; 1 (1/8, 12.5%) and 2 (2/25, 8%) had subclinical hypothyroidism; 6 (6/8, 75%) and 15 (15/25, 60%) had euthyroidism; 1 (1/8, 12.5%) and 4 (4/25, 16%) had subclinical hyperthyroidism; and 0 (0/8, 0%) and 3 (3/25, 12%) had overt hyperthyroidism. Among G3 and G4 patients, 3 (3/45, 6.7%) and 9 (9/55, 16.4%) had overt hypothyroidism; 8 (8/45, 17.8%) and 5 (5/55, 9.1%) had subclinical hypothyroidism; 10 (10/45, 22.2%) and 2 (2/55, 3.6%) had euthyroidism; 6 (6/45, 13.3%) and 4 (4/55, 7.3%) had subclinical hyperthyroidism; and 18 (18/45, 40%) and 35 (35/55, 63.6%) had overt hyperthyroidism. There were no G1 patients with overt hyper- or hypothyroidism. The most common thyroid status among G3 and G4 patients was overt hyperthyroidism. When we compared US grades according to thyroid status, the more severe the thyroid dysfunction, the higher the US grade (*p* = 0.047). TSH level differed significantly between all four US grades when we independently assessed hyperthyroid (*p* = 0.035) and hypothyroid *(p* = 0.027) statuses. There was linear positive correlation and linear negative correlation between TSH and US grades in hypothyroidism and hyperthyroidism respectively (Figs. 6,7). TPOAb, TRAb, and TGAb titers were not significantly associated with US grade.

**Table 1:** Clinical characteristics according to ultrasound grade.

Variable	Total (N = 133)	G1 (N = 8, 6%)	G2 (N = 25, 18.9%)	G3 (N = 45, 33.8%)	G4 (N = 55, 41.3%)	*P* value
Age (year)	15 ± 3.8	13 ± 5	16 ± 3.3	14 ± 4.2	16 ± 3.4	0.751
Male	31 (23.3%)	2 (25%)	4 (16%)	8 (17.8%)	17 (30.1%)	0.352
Female	102 (76.7%)	6 (75%)	21 (84%)	37 (82.2%)	38 (69.1%)	
Thyroid function						
1		0 (0%)	1 (4%)	3 (6.7%)	9 (16.4%)	
2		1 (12.5%)	2 (8%)	8 (17.8%)	5 (9.1%)	
3		6 (75%)	15 (60%)	10 (22.2%)	2 (3.6%)	0.047
4		1 (12.5%)	4 (16%)	6 (13.3%)	4 (7.3%)	
5		0 (0%)	3 (12%)	18 (40%)	35 (63.6%)	
TSH	8.3 ± 22.4	2.7 ± 2.2	8.7 ± 29	10 ± 26.4	6.9 ± 16.2	
TSH (hypo-euthyroid)	14.79 ± 8.7	3.54 ± 1.90	12.62 ± 35.98	19.80 ± 13.26	23.33 ± 22.70	0.035
TSH (hyper-euthyroid)	8.3 ± 22.4	2.19 ± 1.95	1.89 ± 3.50	0.54 ± 0.96	0.21 ± 0.63	0.027
TGAb	410.81 ± 258.58	96.77 ± 61.4	587.50 ± 1464.28	304.42 ± 498.23	654.58 ± 965.73	0.721
TPOAb	1276.77 ± 822.53	93.8 ± 44.98	1650.90 ± 1533.63	1395.56 ± 1499.08	1966.8 ± 1838.87	0.842
TRAb	23.1 ± 26.9	11.1 ± 0.00	5.82 ± 9.04	19.19 ± 28.77	32.96 ± 26.89	0.913

G, grade; N, number of patients; TSH, thyroid stimulating hormone; TG, antithyroglobulin antibodies; TPO, antithyroid peroxidase antibodies; TRAb, TSH receptor antibodies.

Tables 2 and 3 summarize additional analysis of relationship between US grades and functional thyroid status for HT separately from GD. In both HT and GD, there was no statistically significant difference of US grades and functional thyroid status. In HT, TSH level showed positive correlation according to severity of US grades in hypothyroidism. In GD, there was positive correlation and TRAb titer according to severity of US grades.

**Table 2:** Clinical characteristics according to ultrasound grade in HT patients.

Variable	Total (N = 69)	G1 (N = 8, 11.6%)	G2 (N = 17, 24.6%)	G3 (N = 23, 33.3%)	G4 (N = 21, 30.5%)	*P* value
Age (year)	13.5 ± 3.9	12.6 ± 4.9	15 ± 3.3	13 ± 4.3	13 ± 3.7	0.741
Male	29 (42%)	2 (25%)	2 (11.8%)	4 (17.4%)	21(100%)	0.869
Female	40 (58%)	6 (75%)	15 (88.2%)	19 (82.6%)	0 (0%)	
Thyroid function						
1		0 (0%)	1 (5.9%)	3 (13.1%)	7 (33.3%)	
2		1 (12.5%)	1 (5.9%)	7 (30.4%)	4 (19.1%)	
3		6 (75%)	10 (58.8%)	9 (39.1%)	1 (4.7%)	0.44
4		1 (12.5%)	4 (23.5%)	1 (4.3%)	4 (19.1%)	
5		0 (0%)	1 (5.9%)	3 (13.1%)	5 (23.8%)	
TSH		2.65 ± 2.16	12.72 ± 35.95	16.26 ± 31.57	16.01 ± 22.49	
TSH (hypo-euthyroid)	17.92 ± 31.76	3.06 ± 2.05	17.72 ± 42.69	19.21 ± 34.23	23.65 ± 23.15	0.039
TSH (hyper-euthyroid)	2.94 ± 8.64	2.24 ± 1.94	2.82 ± 4.08	1.11 ± 0.22	6.68 ± 18.80	0.631
TGAb	519.50 ± 1161.05	96.76 ± 61.40	715.13 ± 1730.91	438.55 ± 640.39	869.44 ± 1218.19	0.829
TPOAb	1668.99 ± 1636.79	16.533 ± 23.52	1928.44 ± 1573.68	1771.47 ± 1705.83	2454.05 ± 2116.75	0.723
TRAb	13.68 ± 27.93	11.1	5.2 ± 6.81	20.98 ± 37.39	9.39 ± 16.90	0.627

G, grade; N, number of patients; TSH, thyroid stimulating hormone; TG, antithyroglobulin antibodies; TPO, antithyroid peroxidase antibodies; TRAb, TSH receptor antibodies.

**Table 3:** Clinical characteristics according to ultrasound grade in GD patients.

Variable	Total (N = 64)	G1 (N = 0, 0%)	G2 (N = 8, 12.5%)	G3 (N = 22,34.4%)	G4 (N = 34, 53.1%)	
Age (year)	16 ± 3.1		17 ± 2.2	15 ± 3.8	17 ± 2.5	0..862
Male	23 (36%)		2 (25%)	4 (18.2%)	17 (50%)	0.951
Female	41 (64%)		6 (75%)	18 (81.8%)	17 (50%)	
Thyroid function						
1		0 (0%)	0 (0%)	0 (0%)	2 (100%)	
2		0 (0%)	0 (0%)	2 (%)	1 (%)	
3		0 (0%)	5 (%)	1 (%)	1 (%)	0.39
4		0 (0%)	0 (0%)	5 (%)	0 (0%)	
5		0 (0%)	3 (%)	14 (%)	30 (%)	
TSH		NA	0.49 ± 0.64	0.58 ± 1.73	1.58 ± 6.98	
TSH (hypo-euthyroid)	9.44 ± 13.79	NA	NA	4.01 ± 3.18	13.46 ± 17.99	NA
TSH (hyper-euthyroid)	0.13 ± 0.39	NA	0.49 ± 0.64	0.05 ± 0.11	0.08 ± 0.39	0.637
TGAb	338.88 ± 636.41	NA	332.43 ± 716.86	166.52 ± 179.87	460.12 ± 817.11	0.569
TPOAb	1433.10 ± 1579.12	NA	1266.33 ± 1476.47	1224.47 ± 1476.80	1652.51 ± 1710.85	0.753
TRAb	30.64 ± 27.84	NA	1.7 ± 1.06	22.09 ± 28.07	40.54 ± 25.18	0.047

G, grade; N, number of patients; TSH, thyroid stimulating hormone; TG, antithyroglobulin antibodies; TPO, antithyroid peroxidase antibodies; TRAb, TSH receptor antibodies; NA, not assessable.

**Figure 8:**
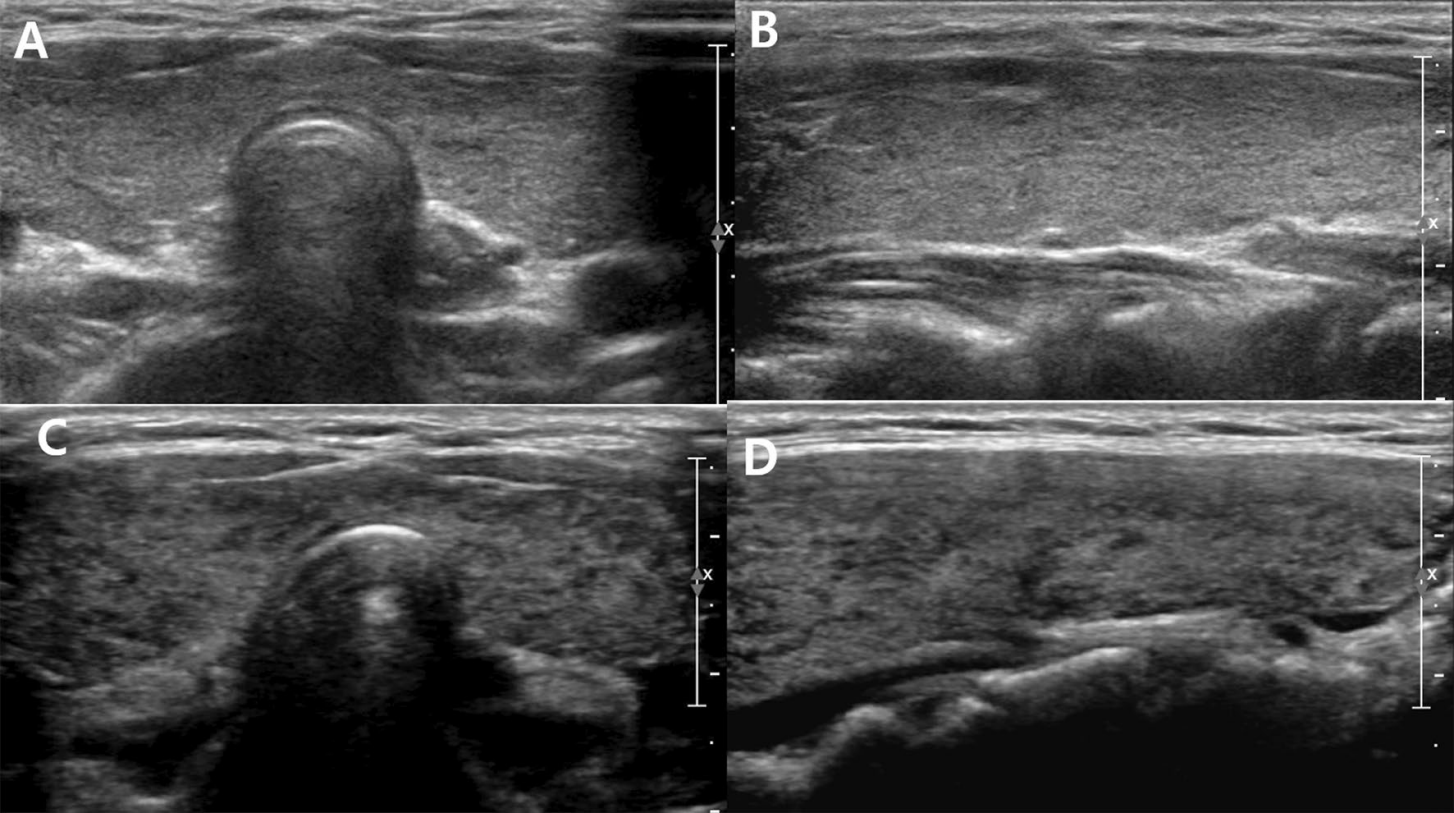
A 16-year-old female patient: initial US (**A** and **B**) was classified as G2. On follow-up US after two years, hypoechogenicity and heterogeneity were aggravated and classified as G4 (**C** and **D**). Simultaneously, TFT results were also aggravated (euthyroidism to overt hyperthyroidism).

**Figure 9:**
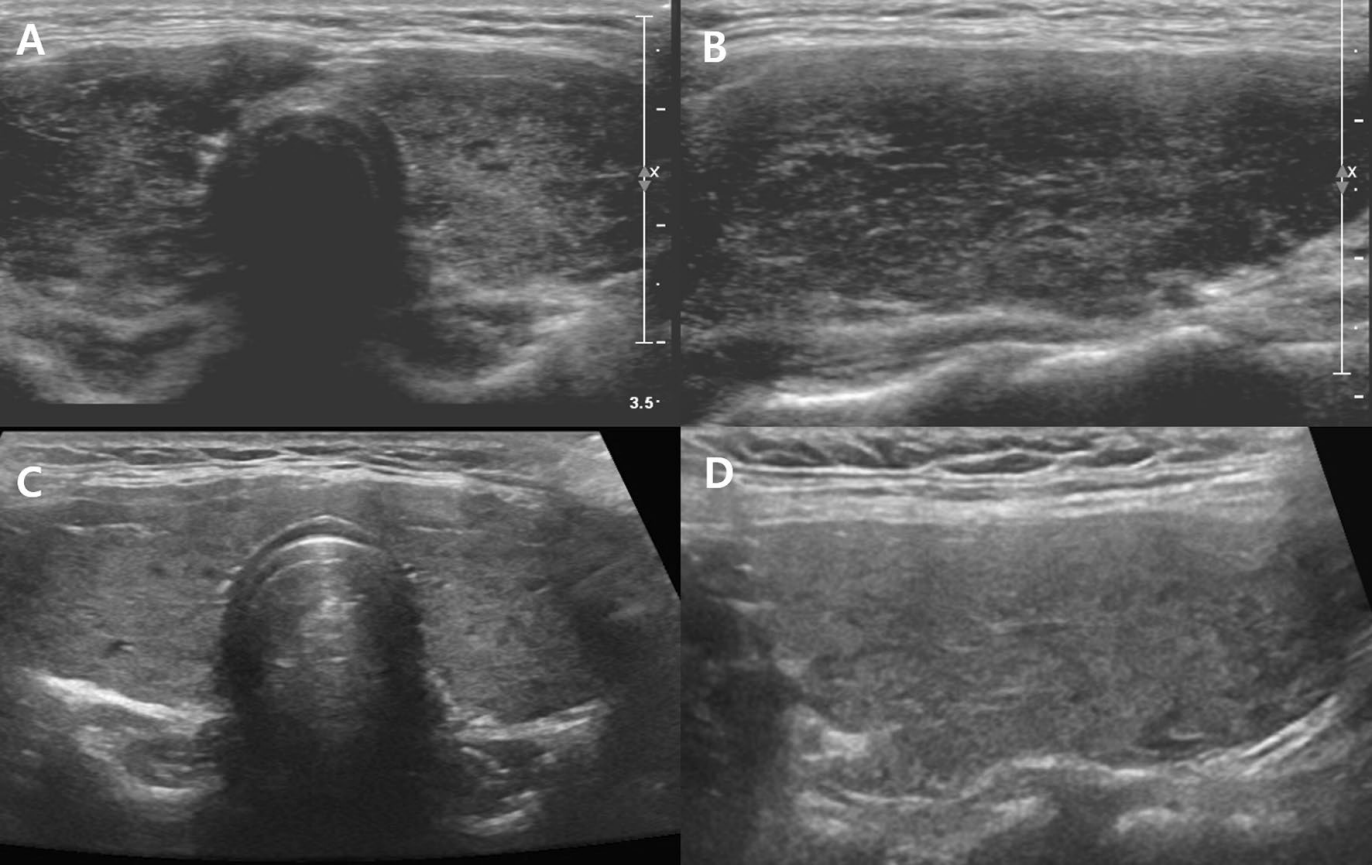
A 14-year-old female patient: initial US (**A** and **B**) was classified as G4. On follow-up US after one year, hypoechogenicity and heterogeneity were improved and classified as G3 (**C** and **D**). Simultaneously, TFT results were also improved (overt hypothyroidism to euthyroidism).

11 patients showed both US grade and TFT changes on follow-up US (Table 4). Among them, six (Fig. 8) showed improvements and five showed aggravation in both thyroid status and US grade (Fig. 9). Among them, three were initial overt hyperthyroidism and two of them became euthyroidism and one became subclinical hyperthyroidism. All three showed improvement on US grade at follow up. All three patients with initial overt hyperthyroidism and hypothyroidism had medication between initial and follow US and thyroid function tests.

**Table 4:** Follow-up US examinations.

No.	Age (years)			Change of US findings (Initial US → follow-up US)		
	Initial US	Follow-up US	AITD	US grade	TFT status	
1	17	18	+ (G)	3 → 2	5 → 3	Improved
2	16	18	+ (H)	3 → 4	3 → 4	Aggravated
3	3	14	+ (H)	3 → 2	2 → 3	Improved
4	16	18	+ (H)	2 → 4	3 → 5	Aggravated
5	14	15	+ (H)	4 → 3	1 → 3	Improved
6	14	14	+ (G)	4 → 3	5 → 4	Improved
7	16	17	+ (G)	3 → 4	4 → 5	Aggravated
8	16	18	+ (G)	3 → 2	4 → 3	Aggravated
9	16	17	+ (G)	4 → 2	5 → 3	Improved
10	5	10	+ (H)	1 → 4	3 → 2	Aggravated
11	9	12	-	3 → 2	4 → 3	Improved

US, ultrasound, H, Hashimoto’s thyroiditis; G, Graves’ disease.

## Discussion

Our study revealed that greater thyroid dysfunction severity was associated with higher US grade in child and adolescent AITD patients. TSH level also differed significantly according to US grade.

AITD is the most common cause of thyroid dysfunction in children and adolescents in iodine-sufficient populations, and its representative diseases are HT and GD^11^. HT and GD are the most common causes of hypothyroidism and hyperthyroidism, respectively^3,4,10–14^. HT, i.e., lymphocytic thyroiditis, is a goitrous form of AITD. In HT, autoantibodies break down thyroid gland cells during immune processes^3,12,13^. GD is caused by thyroid gland stimulation by autoantibodies against the TSH receptor on follicular epithelial cells^14^. These antibodies mimic the effects of TSH, causing overproduction and release of thyroid hormone. The typical HT histological features include lymphoplasmacytic infiltration, germinal center formation, follicular destruction, Hurthle cell change, and varying degrees of fibrosis^9,10,12^, while GD is characterized by histopathological hypercellularity, patchy lymphocyte infiltration, little colloid, and scalloping colloid^15^.

US is a diagnostic tool that is widely used as an adjunct to clinical exams for evaluating thyroid size, anatomy, and parenchymal abnormalities^2,16^. On US, healthy thyroid gland echogenicity is homogeneous and higher than that of the surrounding muscle^17^. AITD patients’ thyroids present differently, with characteristic US findings such as variable degrees of thyroid gland enlargement, decreased parenchymal echogenicity, and heterogeneous parenchymal echo pattern. Thyroid gland enlargement is usually diffuse and symmetric. HT may show as poorly defined, patchy hypoechoic areas and micronodular patterns consisting of multiple small (~ 2–6 mm) hypoechoic nodules. In GD, vascularity tends to be increased, while in HT, it is variable^18^.

Tissue echogenicity of the thyroid gland on US depends on the organ’s cellularity and vascularization. Decreased colloid content, lymphocytic infiltration, and increases in intrathyroidal flow result in hypoechoic tissue patterns^17,19,20^. As mentioned above, inflammatory cells infiltrate and destroy the thyroid gland in HT and GD, which can appear as hypoechogenicity on US.

Heterogeneous echogenicity of the thyroid gland is another well-known finding in AITD^21–24^. Heterogeneous echogenicity is caused by multiple structures of different acoustic impedances creating variable echogenicity degrees on US, and the normal representative tissue is muscle^25^. When healthy, organs such as the thyroid gland or liver consist of characteristic cells with little variation, thus showing homogenous echogenicity on US^25^. Considering the pathological findings of AITD, heterogeneity may also appear on US due to infiltration of other cells in the thyroid gland and fibrosis. Although heterogeneity is a well-known finding in AITD, no studies have evaluated the relationship between thyroid dysfunction severity and heterogenicity degree on US.

Thyroid dysfunction can affect child and adolescent growth and development in various ways. Overt hypothyroidism can cause a potentially fatal medical condition with adverse effects on lipid metabolism and cardiovascular function that occurs in about 10% of HT patients. Onset of this condition is insidious and it may not become clinically apparent until symptoms are abundantly developed^26^. Overt hyperthyroidism in children and adolescents is both less common and more severe than in adults^27,28^. Symptoms of hyperthyroidism include impaired neurodevelopmental outcomes and altered skeletal maturation^25^, such as craniosynostosis and advanced bone age in younger children. Additionally, for school-aged children, poor school performance is common, and may cause severe anxiety in patients and their parents^29^.

AITD, a single disease entity, can manifest in various thyroid function statuses. While subclinical and overt hypo- and hyperthyroidism share similar etiologies, the symptoms of the former are nonspecific and signs are typically absent^30^. Therefore, diagnosis and treatment decisions for subclinical hypo- and hyperthyroidism mainly depend on TFT results^30–32^. Subclinical thyroid dysfunction tends to develop into overt thyroid dysfunction^30,31^, and the risk of progression to overt hypothyroidism in subclinical hypothyroid patients is higher in patients with underlying thyroid disease^16^. It is difficult to predict the risk of progression to a more severe state of thyroid dysfunction. There is no agreement on clinical features, numerical values that indicate mild thyroid dysfunction, or prognosis^10,16^. In our study, there was an association between US grade and TSH level, which was consistent with results from a previous study^26^, where younger patients showed a stronger association between decreased echogenicity and higher TSH, and the relationship was stronger when the changes were recent. Hypoechogenicity of the thyroid gland is a common finding in HT. Jeong et al.^10^, reported that the severity of HT varies depending on hypoechogenicity degree. Other studies have reported that changes in US may be an early sign of more severe thyroid dysfunction and initial hypoechogenicity indicates later development of hypothyroidism^4,9^. Those studies focused on adults and mainly dealt with HT. Our study findings indicate that hypoechogenicity and heterogeneity degree on US are correlated with thyroid dysfunction in child and adolescent AITD patients, including those with GD and HT.

In 11 patients in our study, thyroid function status changed in in a positive relationship with US grade, regardless of whether US grade worsened or improved. This is the first reported finding of not only exacerbation but also remission on US, and it suggests that US changes can reflect thyroid dysfunction status with high sensitivity.

We found no significant associations between echogenicity and TPOAb and TRAb levels. A few studies have assessed the correlation between autoantibody level and hypoechogenicity of the thyroid gland, but none found a significant relationship^9^. However, in mild thyroid dysfunction cases, checking the initial TPOAb level early with US can help predict the course of disease and set the treatment direction^32^.

## Conclusion

We found an association between hypoechogenicity and heterogeneity degree and severity of thyroid dysfunction in child and adolescent AITD patients, including those with normal thyroid function, subclinical thyroid dysfunction, or overt thyroid dysfunction. These results suggest that US findings can be used as another parameter to supplement biochemistry results for thyroid status evaluation.
